# Comparative analyses of fecal microbiota in Chinese isolated Yao population, minority Zhuang and rural Han by 16sRNA sequencing

**DOI:** 10.1038/s41598-017-17851-8

**Published:** 2018-01-18

**Authors:** Ming Liao, Yuanliang Xie, Yan Mao, Zheng Lu, Aihua Tan, Chunlei Wu, Zhifu Zhang, Yang Chen, Tianyu Li, Yu Ye, Ziting Yao, Yonghua Jiang, Hongzhe Li, Xiaoming Li, Xiaobo Yang, Qiuyan Wang, Zengnan Mo

**Affiliations:** 1 Department of Reproductive Center, The First Affiliated Hospital of Guangxi Medical University, Nanning, Guangxi 530021 China; 2 Center for Genomic and Personalized Medicine, Guangxi Medical University, Nanning, Guangxi 530021 China; 3 Guangxi Collaborative Innovation Center for Genomic and Personalized Medicine, Nanning, Guangxi 530021 China; 4 Guangxi Key Laboratory of Genomic and Personalized Medicine, Nanning, Guangxi 530021 China; 5 Oncology Department, Nanning Second People’s Hospital, The third Affiliated Hospital of Guangxi Medical University, Nanning, Guangxi 530031 China; 6 Institute of Urology and Nephrology, First Affiliated Hospital of Guangxi Medical University, Nanning, Guangxi 530021 China; 7 Department of Urology, First Affiliated Hospital of Xinxiang Medical College, Xinxiang, Henan 453100 China; 8 Urology Department, Minzu Hospital of Guangxi Zhuang Autonomous Region, Affiliated Minzu Hospital of Guangxi Medical University, Nanning, Guangxi 530001 China; 9 Department of Emergency Surgery, The first Affiliated Hospital of Guangxi Medical University, Nanning, Guangxi 530021 China; 10 Department of Biostatistics and Epidemiology, Perelman School of Medicine, University of Pennsylvania, Philadelphia, PA 19014 USA; 11 Hezhou University, Hezhou, Guangxi 542899 China

## Abstract

The gut microbiome in humans is associated with geography, diet, lifestyles and so on, but its relationship with some isolated populations is not clear. We used the 16sRNA technique to sequence the fecal microbiome in the Chinese isolated Yao population and compared it with the major minority Zhuang and the major ethnic Han populations living in the same rural area. Information about diet frequency and health status and routine serum measurements were collected. The unweighted UniFrac principal coordinates analysis showed significant structural differences in fecal microbiota among the three ethnic groups. Statistically significant differences were observed in the community richness estimator (chaos) and the diversity estimator (Shannon) among the three groups. At the genus level, the fecal samples of the isolated Yao population presented the lowest relative abundance of the *Megamonas* genus, which was potentially related to the high frequency of bean consumption in the diet. Two enterotypes were identified in the overall fecal microbiota in the three populations. In the isolated Yao population, a higher *Bacteroides* abundance was observed, but the *Prevotella* abundance decreased with increased alcohol consumption.

## Introduction

The human gut microbiota is closely associated with human health^1^. Understanding its abundance and diversity relative to different host characteristics is of great importance. The gut microbiota in humans can be affected by factors such as geography, dietary habits, living environment, age, traditions and changing lifestyles^2,3^. However, inconsistent results have been reported, probably due to the large variations in host properties and the habitat-dependent environmental factors across distinct human populations.

The effect of geographic or ethnic differences on microbiota diversity has not been confirmed. As shown in a survey across five northern European countries, the colonic microbiota structures did not present significant differences with respect to geographic origin, age or gender^4^. In another larger survey across six different countries covering Europe, Japan and the USA, the intestinal microbiota variations can be generally stratified to three enterotypes, but the enterotypes were independent of geography or ethnicity^5^. In another survey that included Malawian, Amerindian and USA residents, the phylogenetic composition of fecal microbiota was significantly different between the individuals in different geographies, probably due to dietary differences. Similar results were observed in a Chinese survey that included 314 healthy young adults from seven ethnic origins^6^. A phylogenetically diverse set of gut microbiota may be common among distinct healthy populations, but phylogenetical diversity was mainly clustered by ethnicities/geography and less by lifestyles^6^. These inconsistent results from various studies need to be further clarified by substantially broader cross-cultural sampling^7^.

China has evolved into a multi-cultural society consisting of Han as the major ethnic group and 55 official ethnic minority groups. The Guangxi Zhuang Autonomous Region has the greatest proportion of the Zhuang minority in China and some other minorities as well. One minority, Yao, with a population of approximately 3 million living mainly in southern China^8^, has a unique genetic background and lifestyle^9–11^. One of its subpopulations, Tu Yao, with a population of 3 thousand, only lives in small villages high in the steep mountains or deep in the dense forests in the Hezhou province of Guangxi Zhuang Autonomous Region. Tu Yao is an isolated population because of a lack of traffic and little contact with the outside world. The original natives had no paddy fields, and slash-and-burn cultivation was always inadequate. This is the first study of this special population, and our study focuses on the fecal microbiota in this group. Because the Han population is the major ethnic group in China, and the Zhuang ethnic group is the major minority in Guangxi, we included fecal samples from Han and Zhuang populations in the mountains in the same area as Tu Yao to minimize the influences of genetic background and the environment. We carried out this comparative analysis in these three ethnic groups to characterize the fecal microbiota in Tu Yao and explore its potential relationship with diet, lifestyle and serum biomarkers and then examined the potential enterotypes^12^ and their association with host properties.

## Results

### Baseline characteristics of the samples in the three ethnic groups

A total of 47 Han, 28 Zhuang and 59 Tu Yao (simplified for Yao as follows) were included in our comparative analysis (Table 1). The proportion of males in the three ethnic groups was not statistically significant (P = 0.110). The participants in Yao appeared significantly younger (P = 0.007), lighter (P < 0.001) and shorter (P < 0.001) compared to the Han and Zhuang. Moreover, more people in the Yao group regularly had breakfast compared to the other two groups (P = 0.002). The people in the Han group were more likely to wash their hands before meals (P = 0.013), but were less likely to drink unboiled water (P = 0.008) compared to the other two groups.

**Table 1:** Characteristics of the included samples*.

	Han		Zhuang		Yao		P
N	47		28		59		
Male	14	(29.8%)	14	(50%)	25	(42.4%)	0.110
Age (yr)	50.0	(42.5–59.5)	55.5	(48.3–62.5)	44.0	(34.0–51.0)	0.007
BMI	23.6	(21.0–25.7)	22.9	(19.9–23.9)	21.3	(20.0–23.5)	0.030
weight (kg)	58.2	(51.4–66.5)	58.5	(49.5–63.3)	49.1	(45.8–54.8)	<0.001
height (cm)	157	(154–164)	158	(153–164)	152	(147–157)	<0.001
alcohol consumption							0.654
Never	30	(63.8%)	18	(64.3%)	32	(54.2%)	
Former	4	(8.5%)	1	(3.6%)	4	(6.8%)	
Current	12	(25.5%)	7	(25%)	22	(37.3%)	
cigarret smoking							0.461
Never	37	(78.7%)	17	(60.7%)	39	(66.1%)	
Former	2	(4.3%)	1	(3.6%)	0	(0%)	
Current	7	(14.9%)	4	(14.3%)	12	(20.3%)	
physical activity							0.125
> = 4 h/d	18	(38.3%)	13	(46.4%)	16	(27.1%)	
<4 h/d	25	(53.2%)	12	(42.9%)	39	(66.1%)	
dietary habits							
have regular meals	42	(89.36%)	25	(89.29%)	49	(83.05%)	0.716
have regular breakfast	40	(85.11%)	27	(96.43%)	58	(98.31%)	0.020
drink unboiled water	19	(40.43%)	16	(57.14%)	31	(52.54%)	0.008
wash hands before meals	41	(87.23%)	17	(60.71%)	49	(83.05%)	0.013
wash teeth regularly	45	(95.74%)	28	(100%)	52	(88.14%)	0.110
prevalent taste							
salty	42	(89.36%)	24	(85.71%)	45	(76.27%)	0.233
spicy	42	(89.36%)	27	(96.43%)	54	(91.53%)	0.600
sugary	42	(89.36%)	24	(85.71%)	56	(94.92%)	0.353

*Continual variables were present as median values (25–75th percentile) and p was calculated by Kruskal-Wallis rank-sum test, whereas categorical variables were present as frequencies and proportions, and p was calculated by Fisher’s exact test.

### Alpha-diversity and beta-diversity analysis

**Figure 1:**
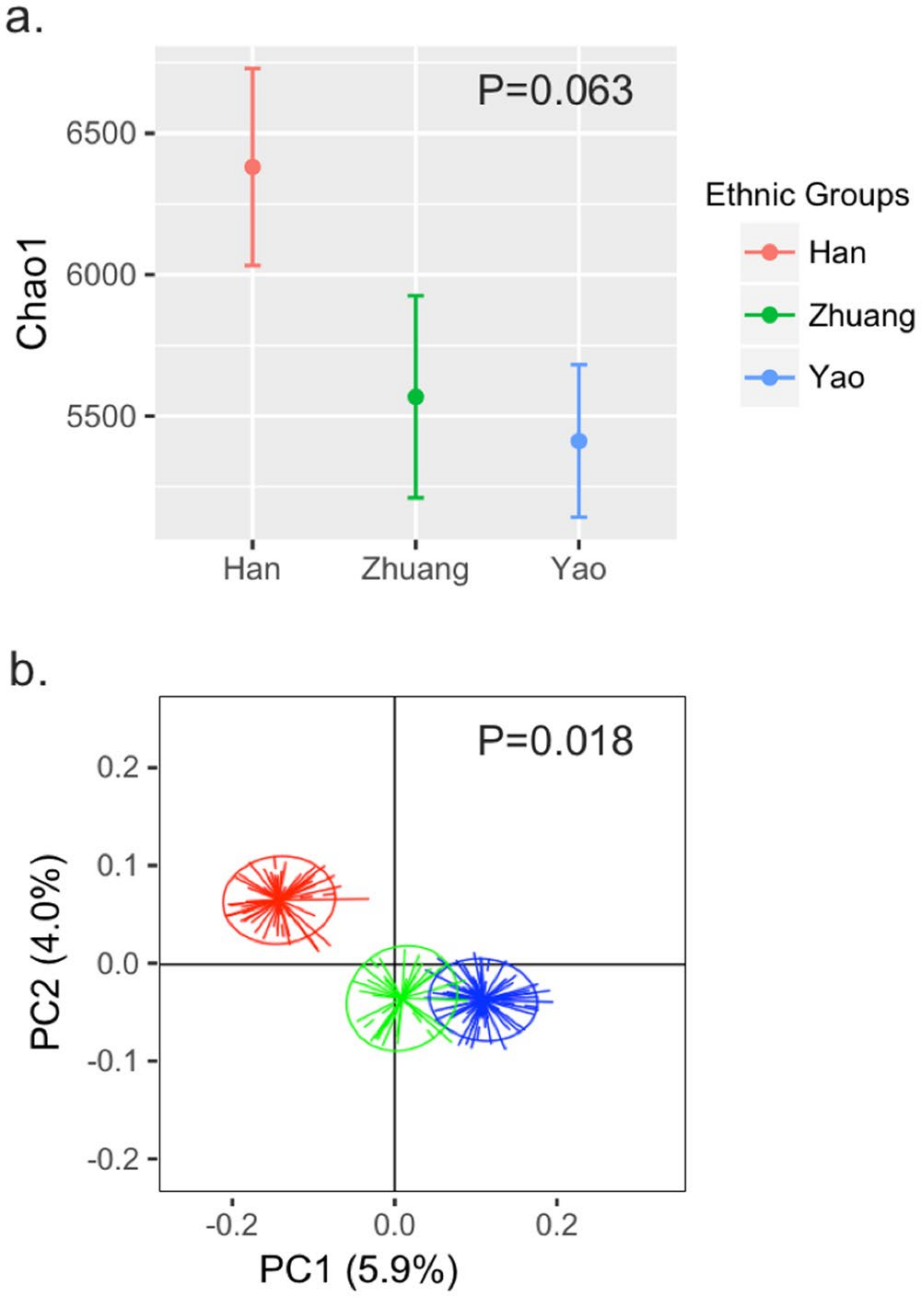
The analysis of alpha-diversity (Chao1 index) and beta-diversity (unweighted Unifrac) in the three ethnic groups. (**a**) The chao1 index at the 4200 sequences per sample in the rarefaction curve; P was calculated from the analysis of variance. (**b**) The unweighted UniFrac; P was calculated from the permutational multivariate analysis of variance.

In total, 3,451,874 raw reads were obtained from all 134 fecal samples. After filtering, 2,270,148 high-quality sequences were produced, with an average of 16,940 ± 4064 clean reads per sample. The total number of OTUs at the 97% similarity level was 687,280. The clean reads were highly correlated with the OTU counts in all samples (Fig. S1). The rarefaction curve of all samples almost reached a plateau at this sequencing depth, suggesting that the sequencing was appropriately deep. Alpha-diversity and beta-diversity were present in the three ethnic groups (Figs 1, S2).The chao1 index appeared to be higher in the Han group than in the other two ethnic groups, but this difference was not statistically significant (Fig. 1a). The MDS for unweighted UniFrac was performed to compare the overall structure of the gut microbiota of all samples based on the relative abundance of OTUs. The PERMANOVA revealed an obvious separation between the Han group and the other two groups (Fig. 1b). A statistically significant compositional difference was observed in the unweighted Unifrac analysis (P = 0.018).

In terms of other estimators of alpha-diversity, the Shannon index appeared to be higher in the Zhuang group compared to the other two groups (P = 0.577), and the index of PD_whole_tree was slightly different between the Yao group and the other two ethnicities (P = 0.432). The observed species among the three groups were almost the same (P = 0.724) (Fig. S2a). Other analysis methods (weighted Unifrac, Bray-Curtis and Jensen-Shannon) for beta-diversity revealed a modest separation between Yao and the other two ethnicities, but this difference was not statistically significant (Fig. S2b).

### Taxonomy-based comparisons at the genus level

**Figure 2:**
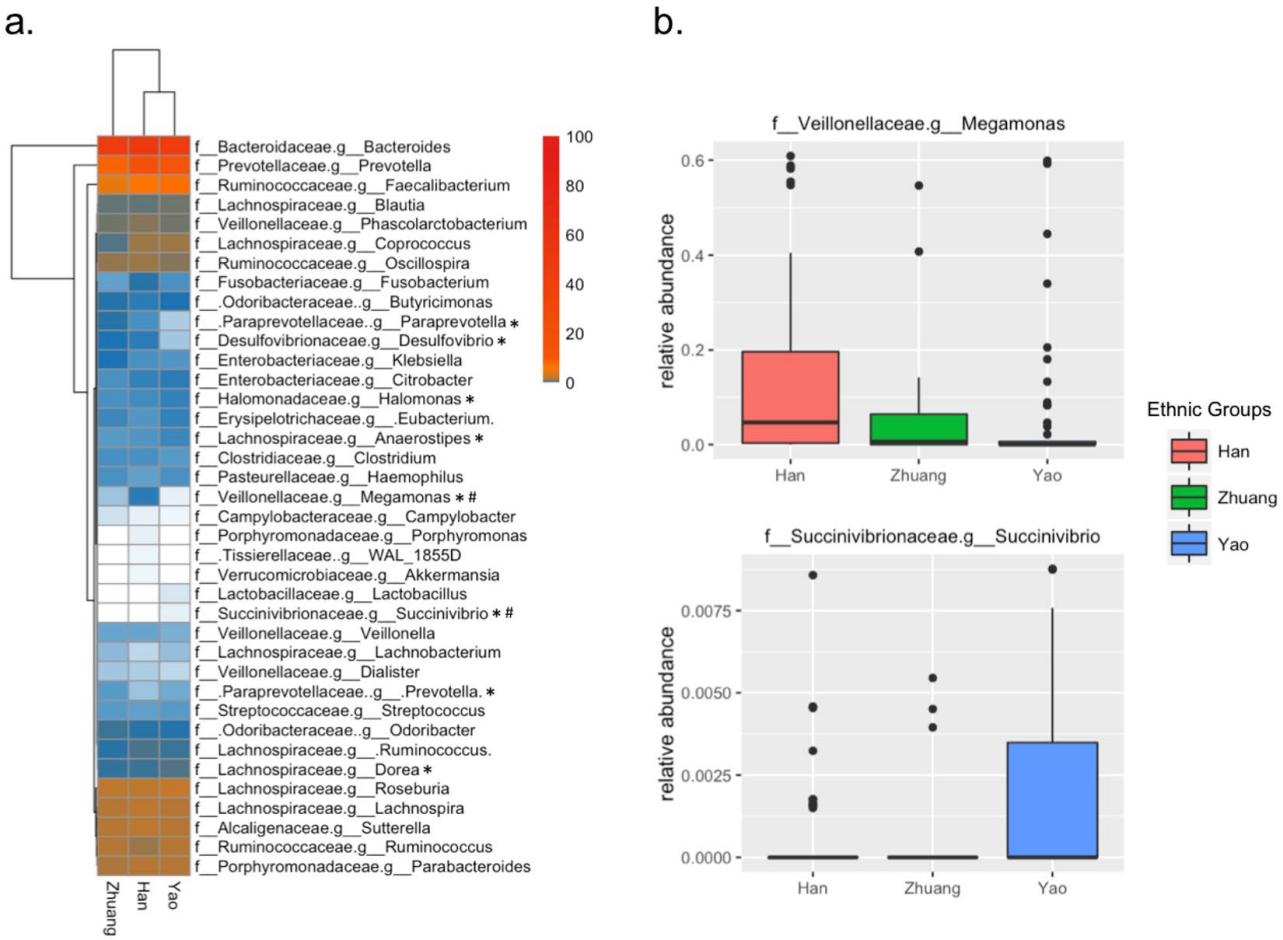
The relative abundance of detected genera in the three ethnic groups. (**a**) The pattern of median values of genus abundance in the three ethnic groups. (**b**) The relative abundance of two significant genera; p was calculated from the Kruskal-Wallis rank-sum test.

The overall microbiota compositions in the three ethnic groups at the family and genus levels are shown in Fig. 2. There were 22 families and 45 genera in the fecal samples. The dominant genera of the three groups were *Bacteroides* and *Prevotella* (Fig. 2a). Eight significant genera were variably distributed in the three groups, but only 2 genera remained statistically significant after Bonferroni adjustments (Fig. 2b). *Megamonas* was significantly more abundant in the fecal microbiota of the Han group compared to the Zhuang (p = 0.048) and the Yao groups (p < 0.001), and *Succinivibrio* was significantly more abundant in the fecal microbiota of the Yao group compared to the Han group (p = 0.054) and the Zhuang group (p = 0.187). The overall presence of microbiota in three ethnic groups at the family and genus levels is shown in Fig. S3. There were three clusters of microbiota, including highly prevalent, modestly prevalent and less prevalent groups. Accordingly, *Megamonas* was highly prevalent, but *Succinivibrio* was less prevalent.

**Figure 3:**
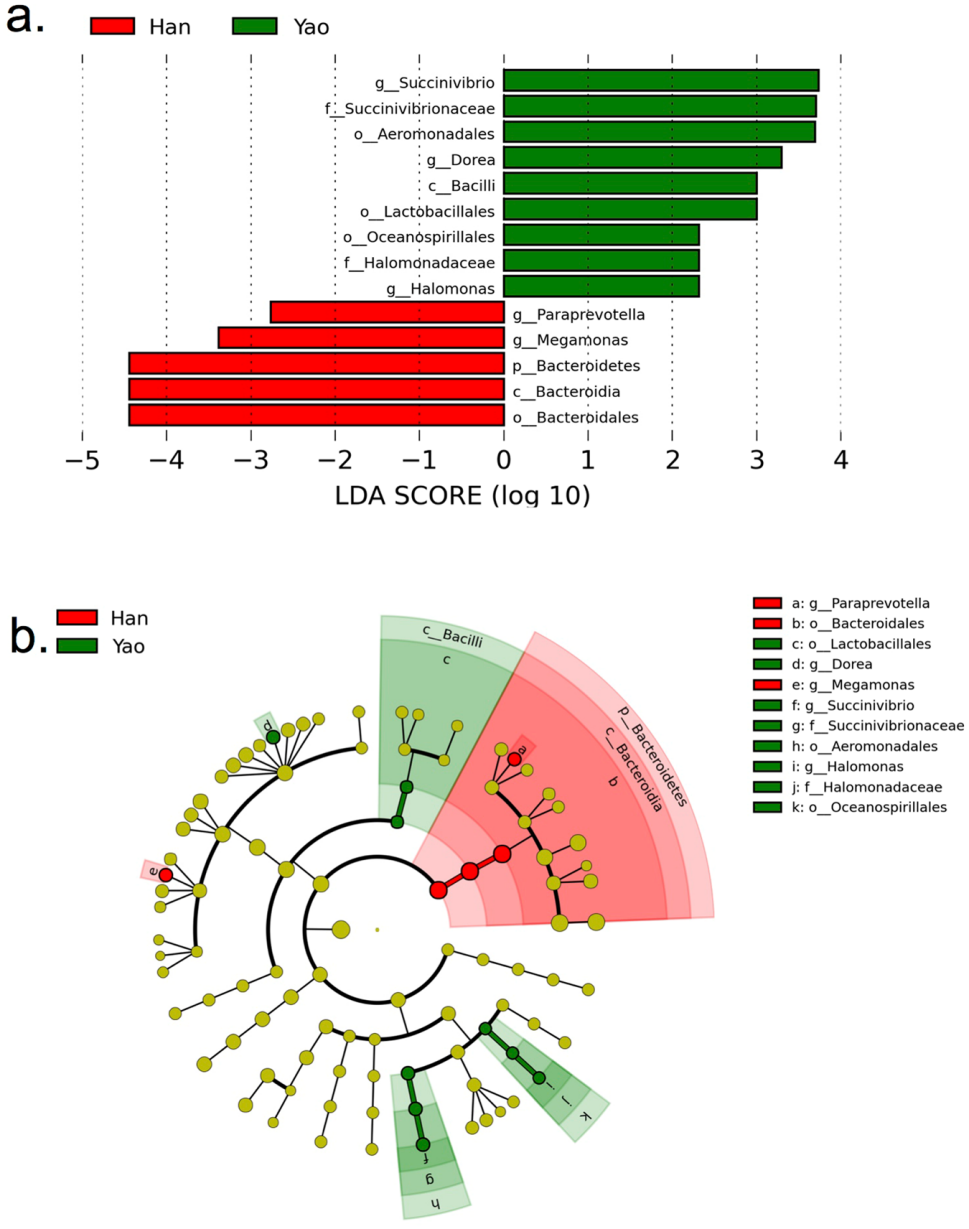
The LEfSe results of the Tu Yao and Han groups. (**a**) The linear discriminant analysis. (**b**) The cladograms report. Prefixes represent abbreviations for the taxonomic rank of each taxa: phylum (p__), class (c__), etc.

We also used the LEfSe algorithm to identify the specific taxa that are variably distributed in the three ethnic groups. By specifying ethic statuses as distinct classes and using the default LDA cutoff of + /−2.0, we found that 5 taxa were over-represented (including the genus *Megamonas*) and 9 taxa were under-presented (including the genus *Succinivibrio*) in the Han group compared to the Yao group (Fig. 3). Additionally, 3 taxa were over-represented (including the genus *Megamonas*) and 1 taxa was under-presented in the Han group compared to the Zhuang group (Fig. S4)

### Diet frequency and serum biomarkers in the Yao group

**Figure 4:**
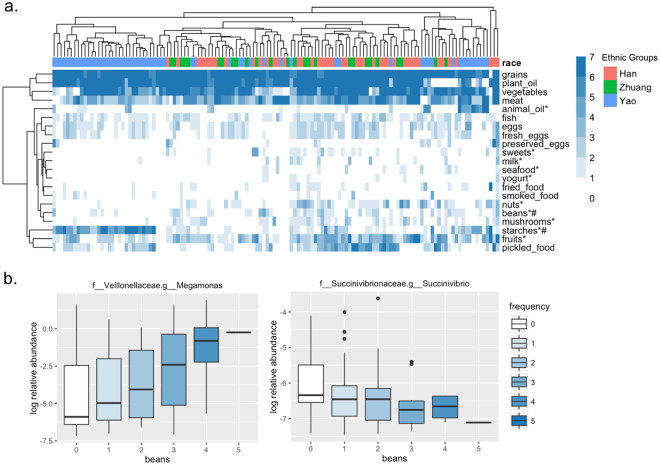
The dietary frequencies in the three ethnic groups. (**a**) The dietary patterns of 21 specific foods in the three ethnic groups; *Indicates p < 0.05 from Fisher’s exact test, ^#^Indicates p < 0.05 after bonferroni adjustments. (**b**) The association of bean consumption frequency with two significant genera; p was calculated from the Kruskal-Wallis rank-sum test.

The diet frequencies of 21 specific foods in all samples are presented in Fig. 4. The diet pattern was slightly different in the Yao group compared to the other two groups (Fig. 4a). The diet frequencies of 10 specific foods were statistically different in all three groups (p < 0.05). The differences in bean consumption frequency remained statistically significant after Bonferroni adjustments, which is potentially related to the relative abundance of either *Megamonas* or *Succinivibrio* **(**Fig. 4b**)**. The *Megamonas* abundance increased (p = 0.034) and the *Succinivibrio* abundance decreased with a higher frequency of bean consumption (p = 0.057).

**Figure 5:**
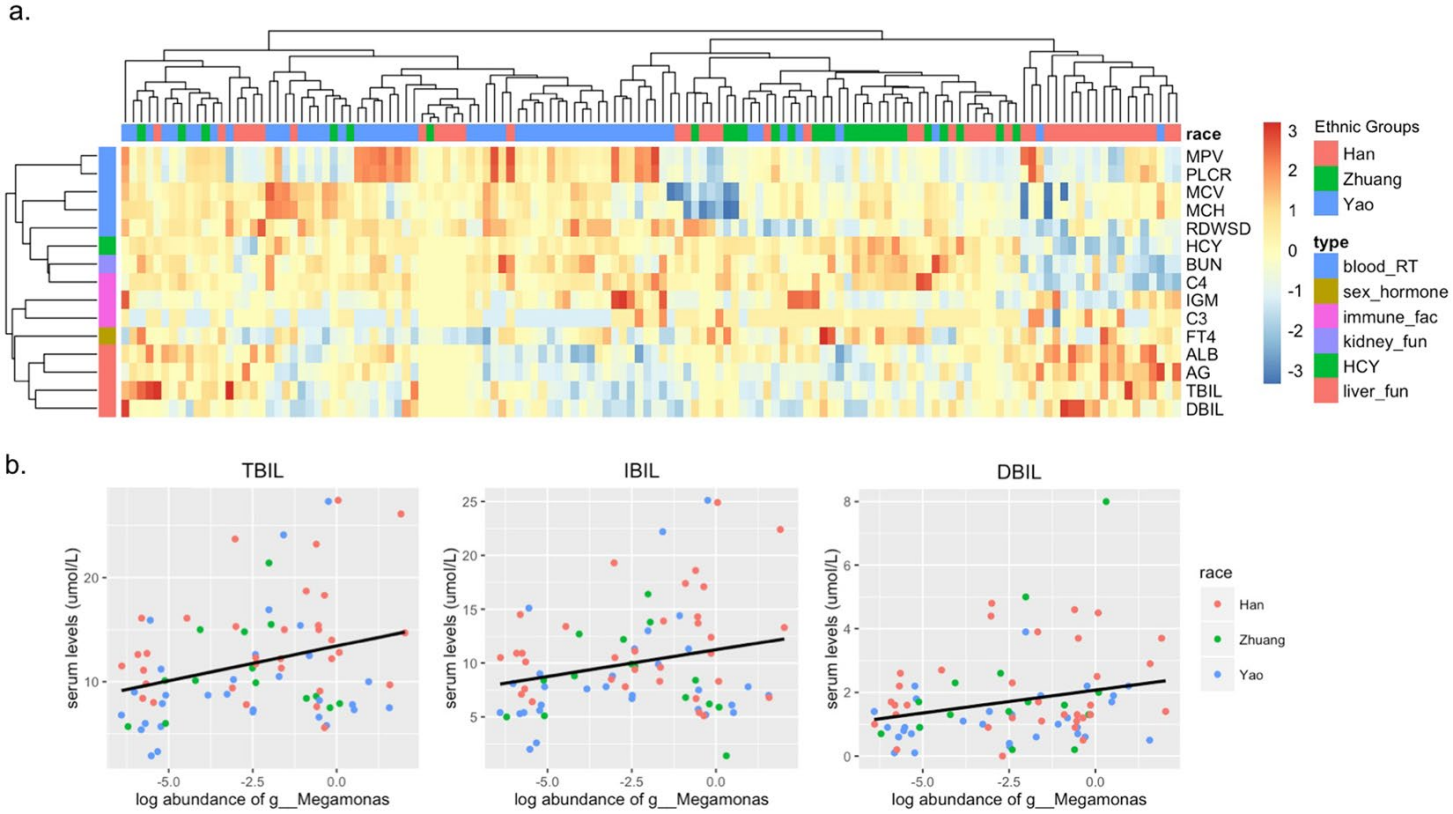
The significant serum biomarkers in the three ethnic groups. (**a**) The distribution pattern of serum biomarkers in the three ethnic groups. Both rows and columns were clustered by *Euclidean* distance, and the serum levels of biomarkers were normalized by z-scores. MPV: mean platelet volume, PLCR: platelet to large cell ratio, MCV: mean cell volume, MCH: mean cell hemoglobin, RDWSD: red cell distribution width - standard deviation, HCY: homocysteine, BUN: blood urea nitrogen, C3: complements 3, C4: complements 4, IgM: immunoglobulin M, FT4: free thyroxin, ALB: albumin, AG: albumin to globulin ration, TBIL: total bilirubin, DBIL: direct bilirubin. (**b**) The association between the serum levels of the three types of bilirubin and the log relative abundance of the *Megamonas* genus; p was calculated from the Spearman correlation analysis. TBIL: total bilirubin (p = ,0.0009 adjusted R-squared = 0.083), IBIL: indirect bilirubin (p = 0.0082, adjusted R-squared = 0.050), DBIL: direct bilirubin (p = 0.0004, adjusted R-squared = 0.094).

A total of 73 serum biomarkers were measured using routine blood tests for liver function, kidney function, sex hormones and immune factors. The predictive accuracy of these biomarkers to distinguish the Yao group from the other two groups was 83% (Fig. S5a). Only 15 biomarkers were significantly different among the three ethnic groups (Figs 5a and S5b). Yao people appeared to have relatively high levels of biomarkers in the routine blood tests, including MPV, PLCR, MCV, MCH and RDW (Fig. S5b). Meanwhile, they appeared to have relatively low levels of biomarkers of liver function, including ALB, AG, TBIL and DBIL (Fig. S5b). The three types of bilirubin were positively correlated to the log relative abundance of the *Megamonas* genus (Fig. 5b).

### Alcohol consumption and the common bacteria in various enterotypes

**Figure 6:**
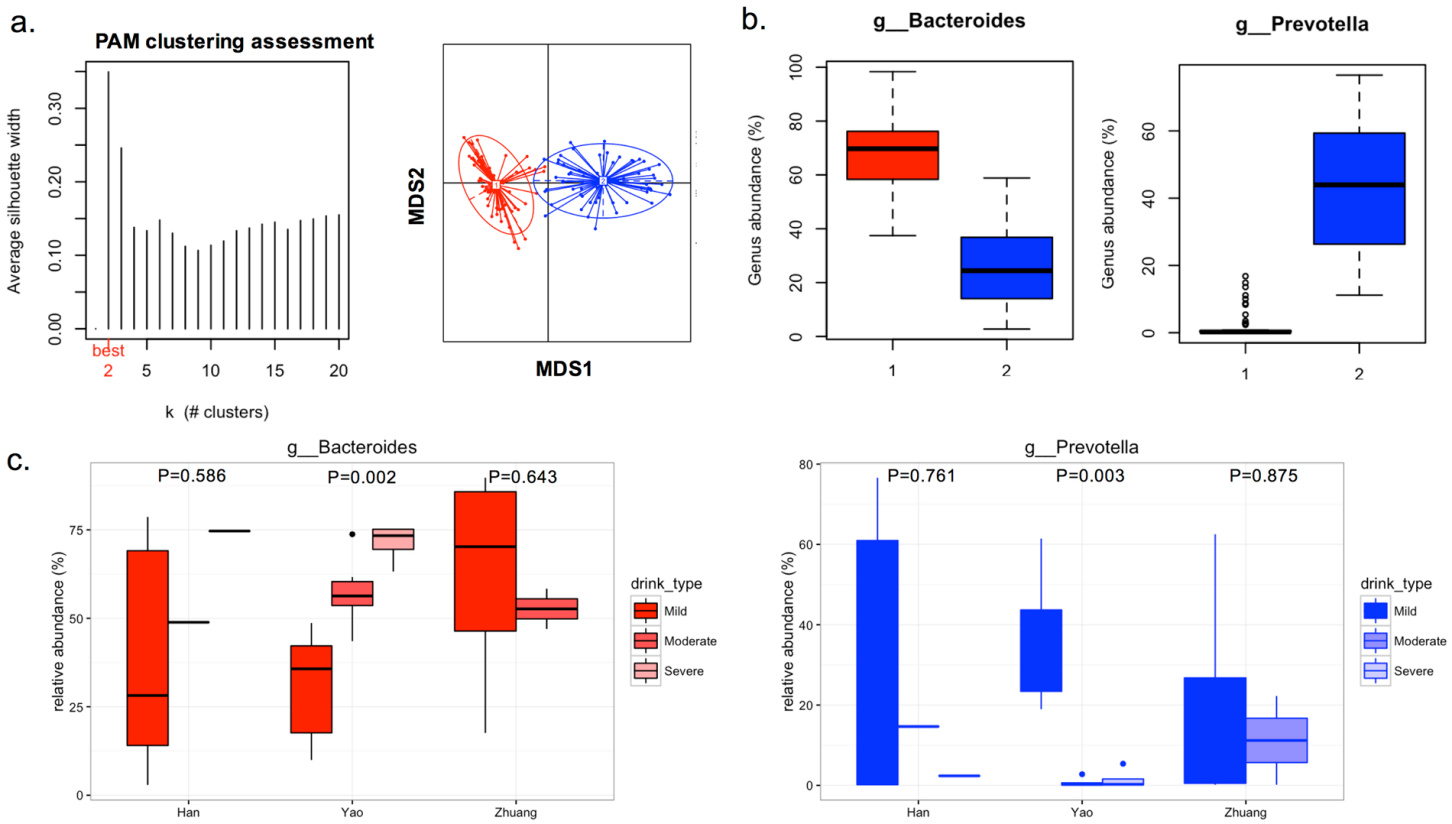
Two common bacteria were associated with alcohol consumption. (**a**) Clustering using the Jensen-Shannon distance in the gut microbiota composition determined by 16 S rRNA gene sequencing. The left panel shows that the data are naturally separated into two clusters by the PAM method. The x-axis shows the cluster number; the y-axis shows silhouette width, a measure of cluster separation. The right panel shows the clustering on the two results from the multidimensional scaling (MDS) analysis. **(b)** The relative abundance of common bacteria in each enterotype. Boxes represent the interquartile range (IQR), and the line inside represents the median. Whiskers denote the lowest and highest values within 1.5 × IQR. **(c)** The association between alcohol consumption and the relative abundance of the two common bacteria in the enterotypes in the three ethnic groups; p was calculated from the Kruskal-Wallis rank-sum test.

The PAM analysis of the Jensen-Shannon distance favored partitioning into two clusters (Fig. 6a**)** but with quite low support (silhouette score 0.3), suggesting that clustering could be due to chance. The two clusters were distinguished by relatively high levels of the genera *Bacteroides* and *Prevotella* (Fig. 6b). The *Prevotella* cluster was distinguished by the additional presence of *Butyricimonas*, *Coprococcus* and *Paraprevotella* (Data not shown). The relative abundances of the *Bacteroides* and *Prevotella* genera were potentially associated with alcohol consumption in both Han and Yao populations, with greater alcohol intake corresponding to a higher abundance of *Bacteroides* and a lower abundance of *Prevotella* (Fig. 6c**)**.

## Methods

### Sampling and information collection

We only enrolled the adults living in Babu district, Hezhou province, Guangxi Zhuang Autonomous Region. The Tu Yao population, one subpopulation of the Yao ethnicity, primarily lives in the villages of Etang town, high up in the mountains with little outside contact. Both the Han and Zhuang populations were living down the mountains in the same area. A comprehensive demographic and health survey was conducted in the form of routine physical examinations from June 2015 to July 2015. All participants included in our analysis were confirmed to have the same corresponding ethnicities within three generations. They had not taken any antibiotics or probiotics in the six months before the sampling dates. A fecal sample was collected from each subject. Fecal samples were frozen immediately after sampling and stored at −80 °C. Ultimately, we enrolled 47 Han, 28 Zhuang and 59 Yao adults in the current study. All the participants provided written informed consent. The survey received the approval from the ethics committee at Guangxi Medical University. All the experiments were performed in accordance with the approved guidelines and regulations.

Information on anthropometric measurements, lifestyles, dietary habits and health status were collected for each subject. The anthropometric measurements, including height and weight, were carried out by well-trained physicians from Guangxi Medical University. Weight was measured with the participants in light clothes but without shoes or heavy accessories using a digital weighing device. Height was measured without shoes, with the buttocks and the heels in close contact with the vertical board, and the movable headboard was placed on the top of the head with sufficient pressure to compress the hair. Body mass index (BMI) was calculated from weight in kilograms divided by height in meters squared. A standardized questionnaire was completed by the same physician after a comprehensive examination and a face-to-face interview with each participant to collect information about smoking status, alcohol consumption, dietary habits, and health status. Both cigarette smoking status and alcohol intake status were defined as never, former (cessation of smoking or drinking for at least six months), and current (daily smoking or drinking within six months). Physical activities were measured by total daily activities; those who regularly exercised for more than 4 h/d were considered physically active. The frequencies of having regular meals or breakfast, drinking unboiled water, washing hands before meals, and brushing teeth regularly were coded as never, sometimes, most times and every day. The taste preferences included salty, spicy or sugary. The diet frequencies of 21 kinds of foods were coded from 1 to 6, indexed as <1 time/month, 1–3 times/month, 1–3 times/week, 4–6 times/week, once/day, and 1–3 times/day respectively.

### Serum assay

Briefly, approximately 10 mL venous blood specimens were obtained between 8 AM and 10 AM after an overnight fast and transported frozen to the Department of Clinical Laboratory at the First Affiliated Hospital of Guangxi Medical University in Nanning on the same day. Serums were centrifuged within 15–25 min and stored at −80 °C until analysis. The Beckman Coulter LH780 Hematology Analyzer was used for routine blood tests and the tests for serum lipids; liver function and kidney function were performed on the Hitachi 7600 auto-analyzer from Japan. Serum sex hormones, immunoglobulins, complements and other immune factors were measured on the COBA 6000 system E601 immunoassay analyzer (Roche diagnostics, IN, Germany).

### DNA extraction, Massively Parallel Sequencing and Sequence Processing

Total DNA was extracted from the pellet using the QIAamp® Fast DNA Stool Mini Kit (QIAGEN) according to the manufacturer’s protocol. The amount of DNA was determined by Nanodrop ND-2000 (Nanodrop, USA). The integrity and the size of the DNA were checked by 1% (w/v) agarose gel electrophoresis. All DNA samples were stored at −20 °C until further processing. Bacterial 16S rDNA amplification and library construction were performed according to the 16S Metagenomic Sequencing Library Preparation guide from Illumina (Forest City, CA) with minor modifications. Briefly, 2 μl of total DNA was amplified using primers targeting the 16S V3 and V4 regions^13,14^ (Illumina). Pooled V3-V4 amplicon libraries were sequenced using the Illumina MiSeq platform with a V3 reagent kit. As an added quality control measure, the software package MacQIIME (version 1.9.1) pipeline^15^ was used to filter out and discard poor-quality reads using the default settings.

### Bioinformatic and Statistical Analyses

Processed sequences were subjected to subsampled, open-reference, operational taxonomic unit (OTU) picking against Greengenes (version 13.8), with unmatched reads clustered by 97% identity into OTUs using UCLUST (http://drive5.com/usearch/manual/uclust_algo.html). Taxonomies were assigned using the UCLUST consensus taxonomy assigner, and a phylogenetic tree was built using FastTree 2.1.3 (http://meta.microbesonline.org/fasttree/). MacQIIME (version 1.9.1) was used to calculate the parameters of alpha diversity, including observed species, chao1, shannon and phylogenetic diversity whole tree (PD_whole_tree), and the parameters of beta diversity, including weighted and unweighted Unifrac distances. The Bray-Curtis distance and the Jensen-Shannon distance were also calculated. The multidimensional scaling (MDS) and the permutational multivariate analysis of variance (PERMANOVA) analyses of the three ethnic groups were performed in R (version 3.2.2)^16^.

To determine which taxa were most likely to explain the differences between the three ethnic groups, taxa summaries generated in MacQIIME were reformatted for input into LEfSe via the Huttenhower Lab Galaxy Server (https://huttenhower.sph.harvard.edu/galaxy/root). This algorithm performed nonparametric statistical testing of whether individual taxa differed between the Han and Yao groups or the Han and Zhuang groups and then differentially ranked the abundant taxa by their linear discriminate analysis (LDA) log-scores. Differentially abundant taxa in the two corresponding groups that were statistically significant using an alpha of 0.05, and LDA log-scores exceeding +/−2.0 were visually represented as bar plots.

The median values of taxa abundance and the median percentages of taxa presence in the three ethnic groups were calculated, and the Manhattan distances were used for the clustering analysis. The Kruskal-Wallis rank-sum test was used to identify significant taxa abundance and Fisher’s exact test was used identify significant taxa presence in the three ethnic groups. Standardized serum biomarkers in the three ethnic groups were compared by the Kruskal-Wallis rank sum test. Random forest (RF) classification was used to evaluate the predictive accuracy of all the serum biomarkers to distinguish the three ethnic groups. Following the suggestion by Arumugam *et al*. that the human gut microbiome can be divided into enterotypes^5^, the partitioning around medoids (PAM) analysis was used to examine the potential clusters in our samples.

## Discussion

Growing evidence supports the diversity of the gut microbiome composition in different ethnic groups. We are the first to focus on one specific group, the Yao, who live in high mountains isolated from the outside world. However, this population is being gradually affected by modern society due to the paving of new roads in the region. Compared to the other two local ethnic groups, the Yao population had a significantly low abundance of the *Megamonas* genus, which may potentially be related to different dietary patterns or different patterns of serum biomarkers. Additionally, the common bacteria *Bacteroides* and *Prevotella* were potentially associated with alcohol consumption.

Studies on the gut microbiomes in different ethnic or demagogic groups have been conducted elsewhere in China, but no studies have focused on the isolated Yao population, which is on the edge of extinction. In a recent survey of 314 healthy young adults living in 9 provinces throughout China, the phylogenetical diversities of the gut microbiota in different ethnic groups were classified^6^. However, the study did not explore the specific genera or species in each ethnic group or examine the potential associations with diet or serum biomarkers. In our current study, both the alpha and the beta diversities in the Han population showed different patterns compared to the other two ethnic groups. The three ethnic groups can be significantly clustered by unweighted UniFrac distance. Moreover, we discovered two significant genera distributed variably in the three ethnic groups. The *Succinivibrio* genus was distinctive in the Yao group, which was in the g:f *Succinivibrionaceae*, h:o_*Aeromonadales*. The *Megamonas* genus was distinctive in the Han group compared to the Zhuang and Yao groups, with a significantly low abundance in the Yao population. We further discovered that the relative abundance of the *Megamonas* genus may be related to the frequency of bean consumption and may be associated with serum levels of bilirubin in the three groups.

The relative abundance of the *Megamonas* genus in the gut has been reported in other studies. A higher abundance of the genera *Megamonas* was observed in Chinese populations compared to African populations^17^, with a lower abundance in Chinese centenarians than in younger elderlies^18^. *Megamonas* appeared to be related to some diseases, as its abundance was higher in obese Taiwanese individuals and lower in patients with Behcet’s Disease or lower motor neuron bowel syndrome^19–21^. Moreover, *Megamonas* in vaginally delivered infants (both neonates and 2-month-olds) had more significant enrichment than in cesarean-delivered infants^22^. In our study, the relative abundance of the *Megamonas* genus was lowest in the Yao population compared to other two ethnic groups, which was probably related to specific dietary patterns. As shown in our study, a higher frequency of bean consumption was potentially associated with a higher relative abundance of *Megamonas* but a lower abundance of *Succinivibrio*. The low abundance of *Megamonas* in the Yao population may be related to the low frequency of bean consumption in this specific ethnic group. Since beans can improve serum lipids by increasing the colonic formation of short chain fatty acids^23^, which has been identified to benefit cardiovascular health^24^, the positive association between bean consumption and the *Megamonas* genus discovered in our study may implicate *Megamonas* as a beneficial microbe. Nevertheless, the effect of a single food on the abundance of a single microbe may be very weak, and the sample size in our study may be inadequate to discover the differences in diet consumption and differences in specific microbial groups. Therefore, we could not rule out the possibility that the association between been frequency and *Megamonas* abundance was due to chance.

We also examined the association between serum biomarkers and the gut microbiota. The serum levels of bilirubin, whether direct, indirect or total bilirubin, appeared to be positively and significantly correlated with *Megamonas* abundance regardless of ethnicity. Serum bilirubin levels were reported to be positively associated with the greater consumption of fruits and vegetables. In our study, the frequency of fruits and vegetables consumption was potentially different among the three ethnic groups. The low level of serum bilirubin in the Yao population may be due to a lower frequency of fruits and vegetables consumption. Moreover, our discovery of a positive association between bilirubin levels and *Megamonas* abundance indicated that the effect of different dietary habits on bilirubin levels may be mediated by the *Megamonas* genus. Growing evidence supports serum bilirubin as a novel biomarker in cardiovascular and metabolic diseases^25^, and our findings also support the modification of dietary habits or even the gut microbiome may influence bilirubin levels and potentially predict health statuses in humans.

The identification of gut microbial clusters in different populations was confirmed in our study. Similar to the enterotypes discovered by Arumugam *et al*.^5^, we found two clusters of fecal communities distinguished primarily by the levels of *Bacteroides* and *Prevotella*. One cluster was predominantly *Bacteroides* and the other cluster was predominantly *Prevotella*. The two clusters did not appear to be associated with the three ethnic groups in our study. However, the dominant genus in two clusters was potentially associated with alcohol consumption. In our subgroup analysis, those who consumed more alcohol had a higher relative abundance of *Bacteroides* but a lower abundance of *Prevotella*. *Prevotella*, which is common in non-Westerners who consume a plant-rich diet^26^ or vegetarians in Western populations^27^, is considered a beneficial microbe^28^, but it is also linked with chronic inflammatory conditions^29^. Our finding correlated a lower *Prevotella* level with alcohol consumption, which can induce a chronic inflammatory status, supporting *Prevotella* as a beneficial microbe. *Bacteroides* and *Prevotella* may be anti-correlated in population-level microbiome surveys, in part because they are antagonistic^28^. Nevertheless, the role of diet in the human gut remains to be determined^30^. Collectively, our findings may support the beneficial role of *Prevotella* in alcohol consumption.

In our current study, we conducted a comparative analysis of fecal microbiota in three populations and discovered that *Megamonas* may be the distinctive genus in the isolated Chinese Yao population compared to the other two local ethnic groups, the minority Zhuang and the rural Han. The relative abundance of the *Megamonas* genus was positively associated with the frequency of bean consumption and the serum levels of bilirubin. Our data also suggested that the dominant genera in the potential enterotypes are associated with alcohol consumption. More studies are needed to clarify the association between enterotypes and alcohol consumption in different ethnic groups.

## Electronic supplementary material

supplementary materials
