# Supplementary material for: Comparative analyses of fecal microbiota in Chinese isolated Yao population, minority Zhuang and rural Han by 16sRNA sequencing
Source: Sci Rep. 2018 Jan 18;8:1142. doi: 10.1038/s41598-017-17851-8 (PMC5773753; doi:10.1038/s41598-017-17851-8)
Supplement: Supplementary file 1 — supplementary materials [file 41598_2017_17851_MOESM1_ESM.pdf]

Comparative analyses of fecal microbiota in Chinese isolated Yao population, minority Zhuang and rural Han by 16sRNA sequencing

Ming Liao[1-4], Yuanliang Xie[2-4], Yan Mao[5], Zheng Lu[2-4,6], Aihua Tan[2-4], Chunlei Wu[7], Zhifu Zhang[8], Yang Chen[2-3], Tianyu Li[2-4,6], Yu Ye[2-3,9], Ziting Yao[2-4], Yonghua Jiang[2-4], Hongzhe Li[10], Xiaoming Li[11], Xiaobo Yang[2-4], Qiuyan Wang[2-4], Zengnan Mo[2-4]

1. Department of Reproductive Center, The First Affiliated Hospital of Guangxi Medical University, Nanning, Guangxi 530021, China
2. Center for Genomic and Personalized Medicine, Guangxi Medical University, Nanning, Guangxi 530021, China.
3. Guangxi Collaborative Innovation Center for Genomic and Personalized Medicine, Nanning, Guangxi 530021, China.
4. Guangxi Key Laboratory of Genomic and Personalized Medicine, Nanning, Guangxi 530021, China.
5. Oncology Department, Nanning Second People's Hospital, the third Affiliated Hospital of Guangxi Medical University, Nanning, Guangxi, 530031, China
6. Institute of Urology and Nephrology, First Affiliated Hospital of Guangxi Medical University, Nanning, Guangxi 530021, China
7. Department of Urology, First Affiliated Hospital of Xinxiang Medical College, Xinxiang, Henan 453100, China
8. Urology Department, Minzu Hospital of Guangxi Zhuang Autonomous Region, Affiliated Minzu Hospital of Guangxi Medical University, Nanning, Guangxi 530001, China
9. Department of Emergency Surgery, the first Affiliated Hospital of Guangxi Medical University, Nanning, Guangxi 530021, China.
10. Department of Biostatistics and Epidemiology, Perelman School of Medicine, University of Pennsylvania, Philadelphia, PA 19014, USA
11. Hezhou University, Hezhou, Guangxi 542899, China.

Correspondence: Zengnan Mo, Institute of Urology and Nephrology, First Affiliated Hospital of Guangxi Medical University, NO.22 Shuangyong Road, 530021 Nanning, Guangxi Zhuang Autonomous Region, China. E-mail: zengnanmo@hotmail.com

Figure 1S. The relationship between clean reads and OUT counts in the three ethnic groups.

Figure 2S. The other alpha-diversity and beta-diversity analysis in three ethnic groups. (a) The Shannon index, PD_whole_tree and observed species at 4200 sequences per sample in the rarefaction curve; P was calculated from the analysis of variance. (b) The weighted Unifrac, Bray-curtis and Jensen-Shannon distance; P was calculated from the permutational multivariate analysis of variance.

Figure 3S. The relative proportions of genus presence in the three ethnic groups. (a) The pattern of the median proportions of genus presence. (b) The presence proportions of two significant genera; p was calculated from Fisher's exact test.

Figure 4S. The LEfSe results by comparison of the Zhuang and Han groups. (a) The linear discriminant analysis. (b) The cladograms report. Prefixes represent abbreviations for the taxonomic rank of each taxa: phylum (p__), class (c__), etc.

Figure 5S. The importance and distribution of serum biomarkers in the three ethnic groups. (a) The importance of each biomarker in random forest classification; * indicates the significant biomarkers from the Kruskal-Wallis rank-sum test. (b) The distribution of the significant biomarkers in routine blood tests and liver function; p was calculated from the Kruskal-Wallis rank-sum test. The bars between the groups indicate $p<0.05$ after multiple comparisons following a significant Kruskal-Wallis test.

Figure. 1S

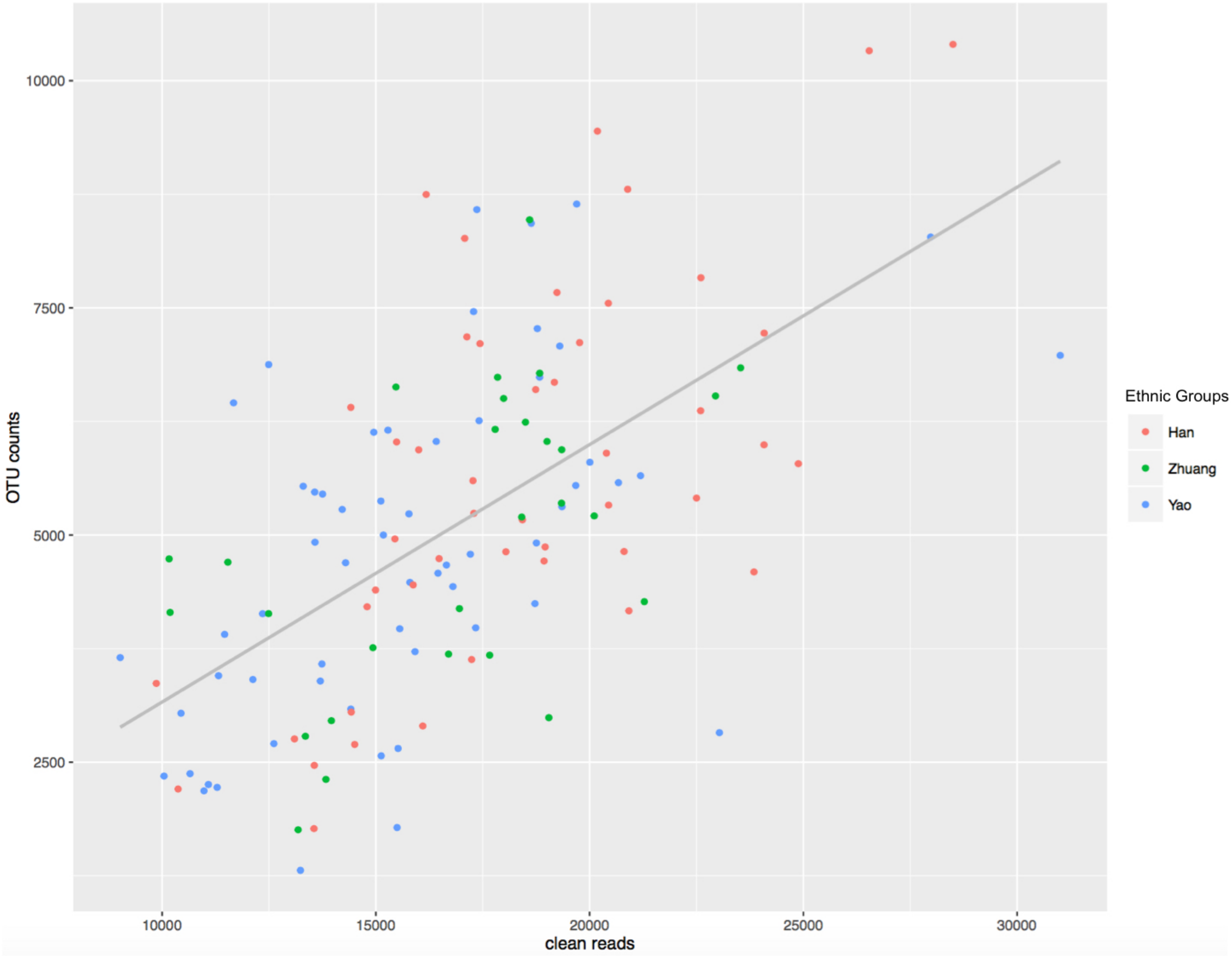

10000
7500
5000
2500
OTU counts
10000
15000
20000
25000
30000
clean reads
Ethnic Groups
Han
Zhuang
Yao

Figure. 2S

a.

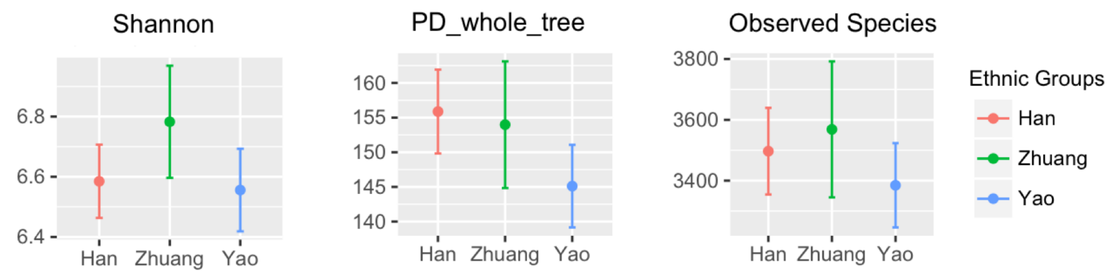

Shannon
6.8
6.6
6.4
Han
Zhuang
Yao
PD_whole_tree
160
155
150
145
140
Han
Zhuang
Yao
Observed Species
3800
3600
3400
Han
Zhuang
Yao
Ethnic Groups
Han
Zhuang
Yao

b.

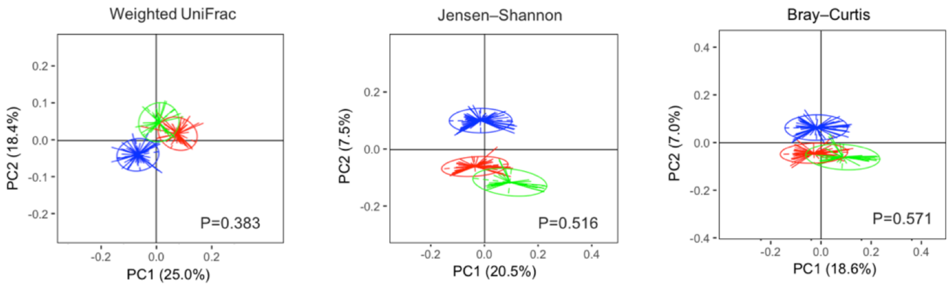

Weighted UniFrac
PC2 (18.4%)
PC1 (25.0%)
P=0.383
Jensen–Shannon
PC2 (7.5%)
PC1 (20.5%)
P=0.516
Bray–Curtis
PC2 (7.0%)
PC1 (18.6%)
P=0.571

Figure. 3S

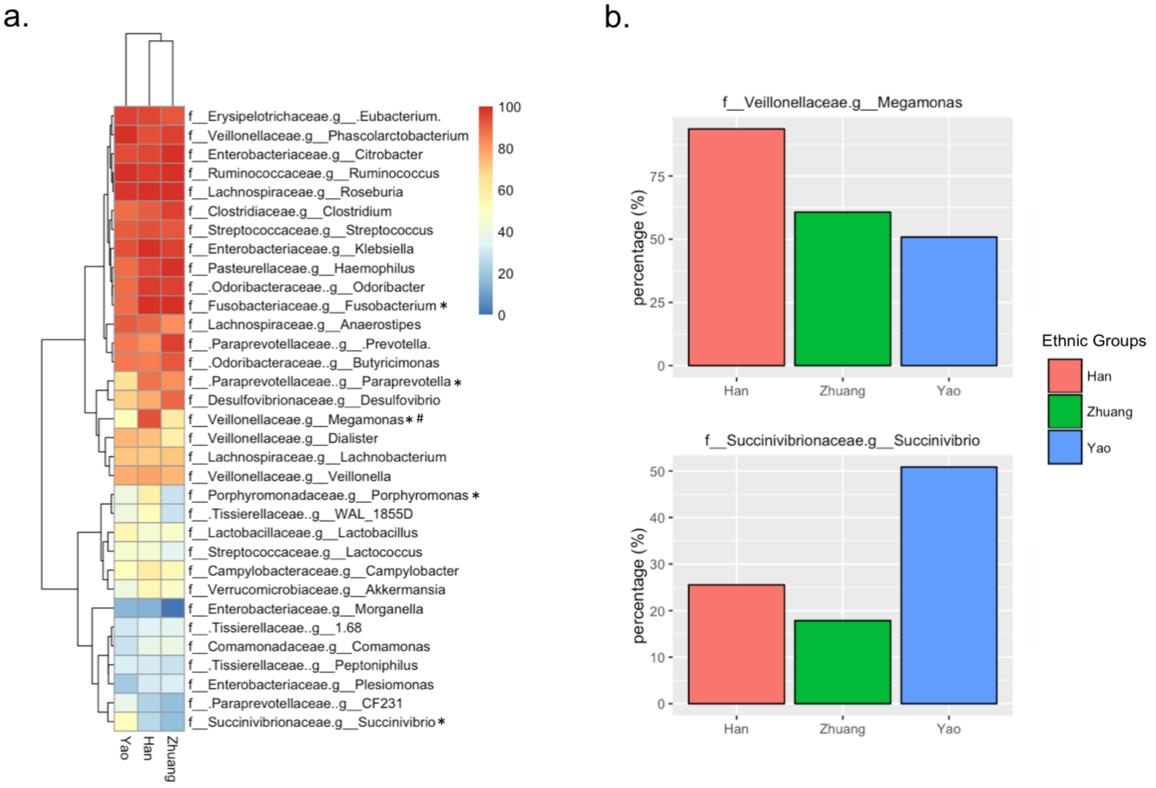
a.
b.
f__Erysipelotrichaceae.g__.Eubacterium.
f__Veillonellaceae.g__Phascolarctobacterium
f__Enterobacteriaceae.g__Citrobacter
f__Ruminococcaceae.g__Ruminococcus
f__Lachnospiraceae.g__Roseburia
f__Clostridiaceae.g__Clostridium
f__Streptococcaceae.g__Streptococcus
f__Enterobacteriaceae.g__Klebsiella
f__Pasteurellaceae.g__Haemophilus
f__.Odoribacteraceae..g__Odoribacter
f__Fusobacteriaceae.g__Fusobacterium *
f__Lachnospiraceae.g__Anaerostipes
f__.Paraprevotellaceae..g__.Prevotella.
f__.Odoribacteraceae..g__Butyricimonas
f__.Paraprevotellaceae..g__Paraprevotella *
f__Desulfovibrionaceae.g__Desulfovibrio
f__Veillonellaceae.g__Megamonas * #
f__Veillonellaceae.g__Dialister
f__Lachnospiraceae.g__Lachnobacterium
f__Veillonellaceae.g__Veillonella
f__Porphyromonadaceae.g__Porphyromonas *
f__.Tissierellaceae..g__WAL_1855D
f__Lactobacillaceae.g__Lactobacillus
f__Streptococcaceae.g__Lactococcus
f__Campylobacteraceae.g__Campylobacter
f__Verrucomicrobiaceae.g__Akkermansia
f__Enterobacteriaceae.g__Morganella
f__.Tissierellaceae..g__1.68
f__Comamonadaceae.g__Comamonas
f__.Tissierellaceae..g__Peptoniphilus
f__Enterobacteriaceae.g__Plesiomonas
f__.Paraprevotellaceae..g__CF231
f__Succinivibrionaceae.g__Succinivibrio *
Yao
Han
Zhuang
100
80
60
40
20
0
f__Veillonellaceae.g__Megamonas
percentage (%)
75
50
25
0
Han
Zhuang
Yao
Ethnic Groups
Han
Zhuang
Yao
f__Succinivibrionaceae.g__Succinivibrio
percentage (%)
50
40
30
20
10
0
Han
Zhuang
Yao

Figure. S4

a.

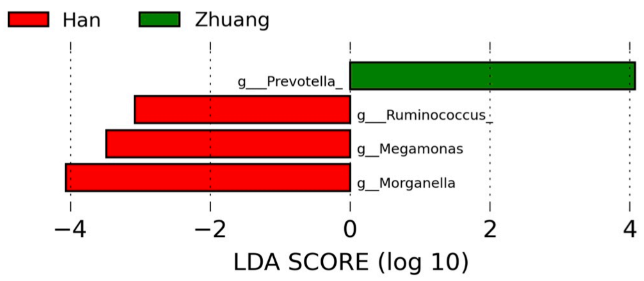
Han
Zhuang
g___Prevotella_
g___Ruminococcus_
g__Megamonas
g__Morganella
−4
−2
0
2
4
LDA SCORE (log 10)

b.

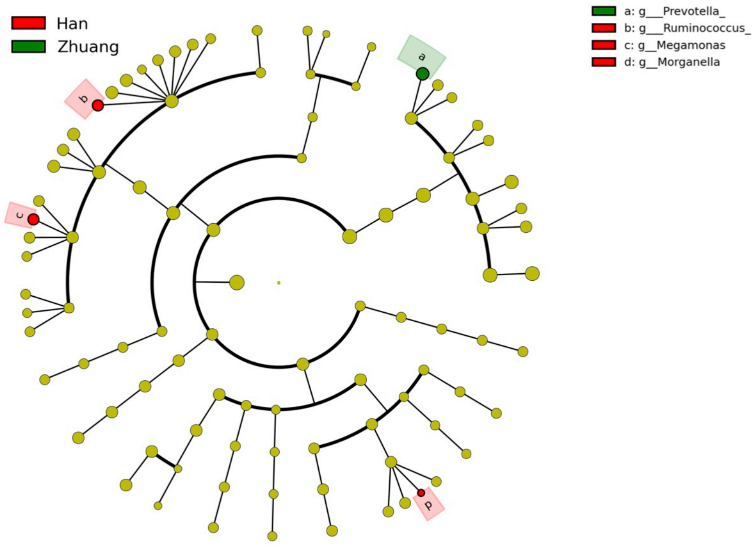
Han
Zhuang
a: g___Prevotella_
b: g___Ruminococcus_
c: g__Megamonas
d: g__Morganella
a
b
c
d

Figure. S5

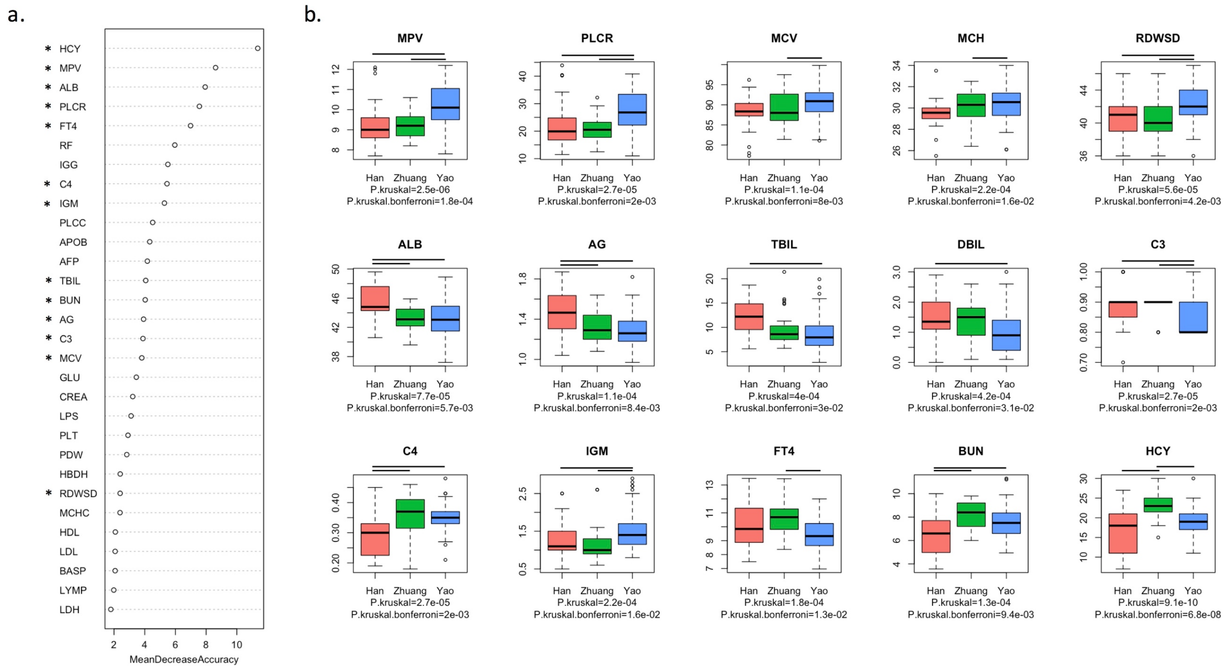
a.
b.
HCY
MPV
ALB
PLCR
FT4
RF
IGG
C4
IGM
PLCC
APOB
AFP
TBIL
BUN
AG
C3
MCV
GLU
CREA
LPS
PLT
PDW
HBDH
RDWSD
MCHC
HDL
LDL
BASP
LYMP
LDH
MeanDecreaseAccuracy
MPV P.kruskal=2.5e-06 P.kruskal.bonferroni=1.8e-04
PLCR P.kruskal=2.7e-05 P.kruskal.bonferroni=2e-03
MCV P.kruskal=1.1e-04 P.kruskal.bonferroni=8e-03
MCH P.kruskal=2.2e-04 P.kruskal.bonferroni=1.6e-02
RDWSD P.kruskal=5.6e-05 P.kruskal.bonferroni=4.2e-03
ALB P.kruskal=7.7e-05 P.kruskal.bonferroni=5.7e-03
AG P.kruskal=1.1e-04 P.kruskal.bonferroni=8.4e-03
TBIL P.kruskal=4e-04 P.kruskal.bonferroni=3e-02
DBIL P.kruskal=4.2e-04 P.kruskal.bonferroni=3.1e-02
C3 P.kruskal=2.7e-05 P.kruskal.bonferroni=2e-03
C4 P.kruskal=2.7e-05 P.kruskal.bonferroni=2e-03
IGM P.kruskal=2.2e-04 P.kruskal.bonferroni=1.6e-02
FT4 P.kruskal=1.8e-04 P.kruskal.bonferroni=1.3e-02
BUN P.kruskal=1.3e-04 P.kruskal.bonferroni=9.4e-03
HCY P.kruskal=9.1e-10 P.kruskal.bonferroni=6.8e-08
Han
Zhuang
Yao
